# YOLO-LS: a novel deep learning framework for brain tumor segmentation in Magnetic Resonance Imaging

**DOI:** 10.1038/s41598-026-48825-4

**Published:** 2026-05-13

**Authors:** Jinghui Chen, Yan Hu, Tao Yang, Zhipeng Sun, Lianxin Xie, Hongjia Zhao

**Affiliations:** 1 The First Clinical Medical College, The Affiliated People’s Hospital of Fujian University of Traditional Chinese Medicine, Fuzhou, Fujian China; 2 The Affiliated People’s Hospital of Fujian University of Traditional Chinese Medicine, Fuzhou, Fujian China

**Keywords:** Brain tumor, Artificial intelligence, Image segmentation, YOLO11, Magnetic resonance, YOLO-LS, Cancer, Computational biology and bioinformatics, Engineering, Mathematics and computing, Medical research

## Abstract

Brain tumors exhibit high heterogeneity in morphology, texture, and location, making accurate recognition and segmentation critical for clinical diagnosis, surgical planning, and prognosis evaluation. However, manual annotation of MRI scans is hindered by subjective bias and inefficiency. Furthermore, existing automated frameworks often face a trade-off between segmentation precision—particularly at infiltrative boundaries—and the computational efficiency required for deployment in resource-constrained environments. To address these challenges, this study proposes YOLO-LS (Lightweight Segmentation), an enhanced framework based on the YOLO11n-seg architecture designed for efficient detection and high-precision segmentation of brain tumors. The methodology introduces three key innovations: (1) integrating ShuffleNet V1 as a lightweight backbone to significantly reduce parameter count and computational complexity via pointwise grouped convolutions; (2) incorporating the DySample dynamic upsampling mechanism to mitigate the loss of fine-grained semantic details inherent in traditional interpolation, thereby improving the recovery of tumor boundaries; and (3) optimizing the neck network with a C3k2-PoolingFormer module to facilitate efficient cross-scale feature fusion and global context capture. The model was trained and tested on the Figshare dataset (3,064 images) using five-fold cross-validation and externally validated on an independent Kaggle dataset (300 images). Results demonstrate that YOLO-LS achieved a bounding box mAP50 of 0.953 ± 0.011, a Dice coefficient of 0.91 ± 0.01, and a 95% Hausdorff Distance (HD_95_) of 4.35 ± 0.34 mm on the internal test set, the latter indicating superior boundary adherence compared to baseline models (paired t-test, *p* < 0.05 for all key metrics). Notably, the model reduced computational cost to 8.1 GFLOPs (a 15.6% reduction) while achieving an increase of 2.9% points in mAP50 compared to the baseline YOLO11n-seg. Comparative analyses with state-of-the-art architectures, including U-Net, SegNet, Swin-UNet, and VM-UNet, confirmed the efficacy of these improvements. Furthermore, Grad-CAM heatmaps validated the model’s precise focus on tumor core and edge regions. On the external test set, the model exhibited strong generalization with a Dice coefficient of 0.895. These findings indicate that YOLO-LS achieves an effective balance between precision, efficiency, and interpretability, demonstrating significant potential for assisting diagnostic workflows in resource-constrained clinical environments.

## Introduction

Brain tumors encompass a diverse group of intracranial neoplasms characterized by rapid progression, neurological impairment, and significant therapeutic challenges^1,2^. Primary tumors, such as gliomas, meningiomas, and pituitary adenomas, exhibit substantial heterogeneity in biological behavior, growth location, and invasiveness^3,4^. For instance, gliomas are associated with a poor prognosis (five-year survival rate of 5–10%) and typically present with infiltrative, blurred boundaries that complicate surgical resection^5^. Consequently, early detection and precise delineation of tumor boundaries are paramount for optimizing surgical strategies, radiotherapy planning, and prognosis assessment. However, traditional manual annotation is labor-intensive and prone to inter-observer variability, failing to meet the demands of large-scale, real-time clinical workflows.

Magnetic Resonance Imaging (MRI), offering multi-sequence high-contrast soft-tissue visualization, serves as the standard modality for brain tumor diagnosis^6,7^. Recently, deep learning has achieved remarkable progress in automating MRI analysis. CNNs, particularly U-Net variants, have established strong baselines: Pourmahboubi et al. improved brain tumor segmentation via transfer learning with pretrained VGG19 weights^8^, and Lu et al. leveraged the Mamba state space model with Fourier-domain feature learning for robust multimodal segmentation^9^. While these CNN-based methods enhance local feature extraction through attention mechanisms or residual connections, they remain limited by fixed receptive fields, hindering the effective capture of long-range dependencies.

To overcome these limitations, Transformer architectures have been introduced to brain tumor segmentation. Jia et al. combined CNNs with Vision Transformers for joint local-global modeling^10^; Zhu et al. integrated multi-physics information fusion for improved multimodal segmentation^11^; and Huang et al. advanced 3D multi-scale attention with deep supervision^12^ and Transformer-based GANs^13^. These Transformer-based methods achieved breakthroughs in global context modeling but suffer from high computational complexity, limiting real-time applications. Recent works have further expanded the scope with novel architectures for enhanced accuracy and multi-modal analysis^14–16^. These studies highlight the continuous need for architectural innovation to address clinical complexities.

The YOLO (You Only Look Once) series, as a representative single-stage object detection framework^17^, has achieved widespread adoption since 2015 for its real-time end-to-end design. Its evolution spans multiple generations: YOLOv1 established the foundational architecture^18^, YOLOv2 introduced anchor boxes and batch normalization^19^, and YOLOv3 enhanced multi-scale target detection via multi-scale predictions and the Darknet-53 backbone^20^. Subsequent versions (YOLOv4–v5) integrated CSPNet and advanced augmentation^21^, while recent versions (YOLOv6–11) explored reparameterization and anchor-free heads, extending to instance segmentation with YOLOv8-Seg and YOLO11-Seg^22^. These models enable pixel-level segmentation while maintaining real-time capabilities, providing efficient solutions for medical imaging tasks.

The YOLO series has been increasingly applied to brain tumor segmentation. Montalbo applied YOLOv4-Tiny with transfer learning for brain tumor detection on CE-MRI^23^. Abdusalomov et al. integrated CBAM and feature pyramid networks into YOLOv7 for brain tumor detection^24^. Yang et al. incorporated ASPP and attention modules into YOLOv5s for tumor segmentation^25^. Abraham et al. enhanced YOLOv8 with dilated convolutions and dual feature pyramid networks for improved small tumor detection^26^. Priyadharshini et al. compared YOLOv9, YOLOv10, and YOLO11 variants on Figshare and BraTS-2020 datasets^27^. Despite these advances, existing YOLO variants for brain tumor segmentation share three key limitations: (1) standard upsampling methods that fail to preserve fine-grained details for small, infiltrative lesions; (2) heavy backbones that hinder lightweight deployment; and (3) insufficient cross-scale feature fusion for handling tumor heterogeneity.

To address the limitations of existing methods, this study proposes YOLO-LS (Lightweight Segmentation). The main contributions of this paper are summarized as follows:

1. Lightweight Backbone: Integration of ShuffleNet V1 to replace the standard CSPDarknet, significantly reducing GFLOPs and parameter count to facilitate suitability for resource-constrained deployment scenarios.
2. Dynamic Upsampling: Adoption of the DySample mechanism to replace nearest-neighbor interpolation, enhancing the recovery of fine-grained details in small tumor lesions.
3. Feature Fusion: Proposal of the C3k2-PoolingFormer module, which utilizes pooling-based token mixing to capture global dependencies without the heavy computational cost of standard Transformers.
4. Comprehensive Validation: Validation of the model using internal and external datasets, employing boundary metrics (HD95) and interpretability tools (Grad-CAM) to ensure clinical reliability.

## Baseline model

**Fig. 1:**
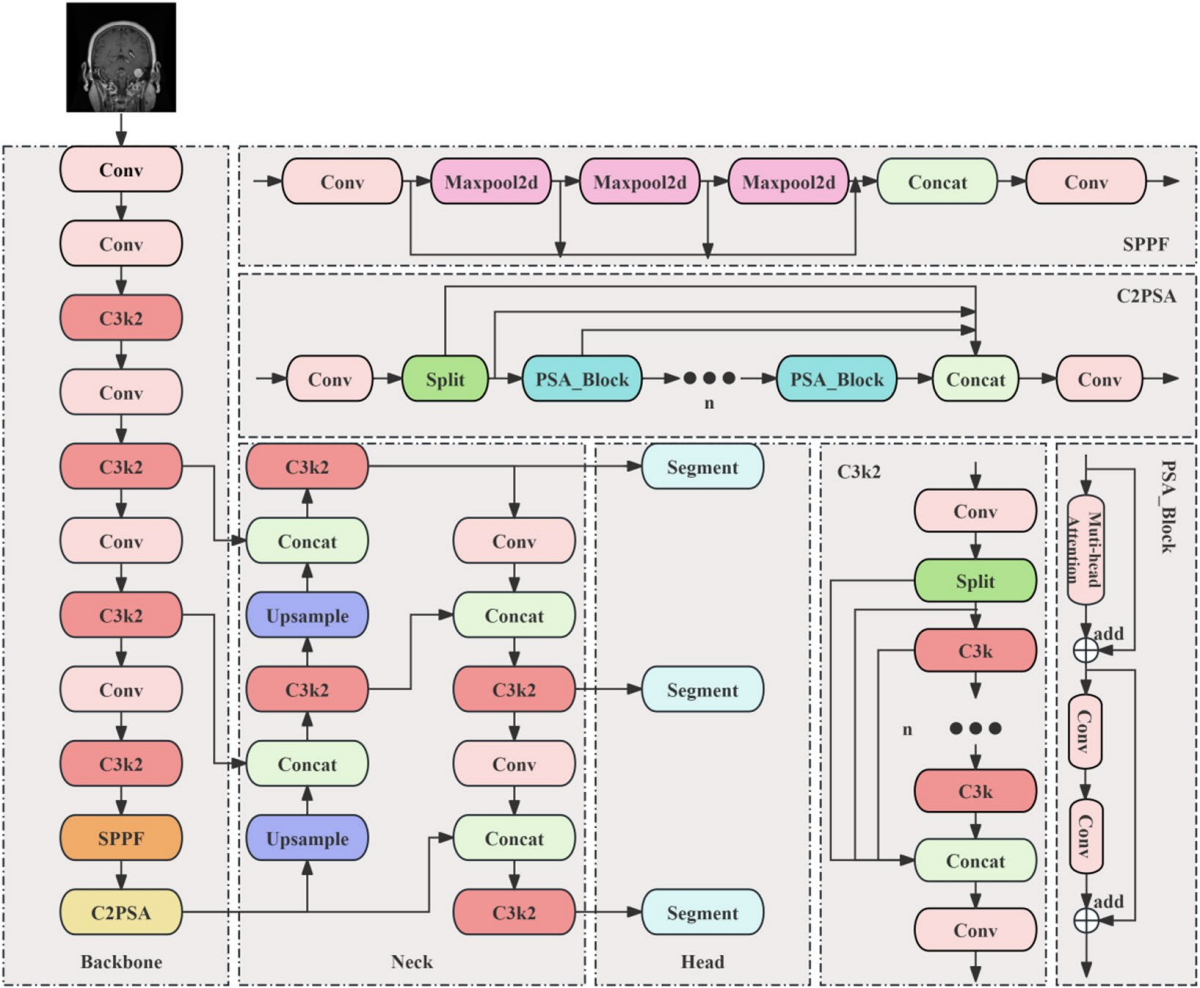
Basic network structure of YOLO11n-seg.

The YOLO series, as a classic algorithm in object detection, is renowned for its real-time capabilities, accuracy, and ease of use. It has been widely extended to instance segmentation tasks, particularly suitable for recognition and precise boundary segmentation of brain tumor MRI images. In clinical scenarios, the end-to-end design of this series enables efficient processing of various MRI modalities, achieving tumor localization, type classification, and pixel-level segmentation to assist in real-time diagnosis and preoperative planning. YOLO11, as a current mainstream algorithm for object recognition, detection, and segmentation, offers five scale variants: YOLO11n, YOLO11s, YOLO11m, YOLO11l, and YOLO11x, accommodating diverse deployment needs from lightweight edge devices to high-performance servers. Its core architecture consists of a backbone network (for multi-scale feature extraction), a neck network (for feature fusion and enhancement), and a head network (for bounding box regression and mask prediction), supporting the YOLO11-Seg variant for direct output of instance segmentation results for tumor regions. Compared to previous versions, YOLO11’s key innovations include the introduction of the C3k2 module as an optimized CSP variant, which optimizes information flow through feature map splitting and small-kernel convolutions to improve computational efficiency; this module supports parameterized configuration for dual-module serialization to increase feature depth or degradation to YOLOv8’s C2f module, indirectly enhancing capture precision of heterogeneous textures in brain tumors; the addition of the C2PSA module fuses CSP structures with partial self-attention (PSA) mechanisms, where features are split via 1 × 1 convolutions—one path passes directly, the other undergoes PSA multi-scale convolution extraction, SE module channel weighting, and Softmax for point-wise weighting—to strengthen focus on key tumor regions, significantly improving detection robustness for multi-scale tumors in complex MRI images; the detection head borrows from YOLOv10 by incorporating depthwise separable convolutions to reduce redundant computations and accelerate inference speed, suitable for resource-constrained clinical workstations. However, the baseline YOLO11 model faces challenges in brain tumor MRI segmentation tasks: high computational costs in the backbone network hinder deployment on edge devices; insufficient feature extraction capabilities lead to loss of tumor details; and low-resolution feature maps struggle to capture small-volume, boundary-ambiguous tumor regions, limiting detection accuracy and segmentation precision. In light of these issues, YOLO11n-Seg is selected as the baseline model for improvements in this paper, with its network structure shown in Fig. 1, laying the foundation for subsequent lightweighting and detail enhancement optimizations tailored to brain tumor characteristics.

## Materials and methods

### Improved model

**Fig. 2:**
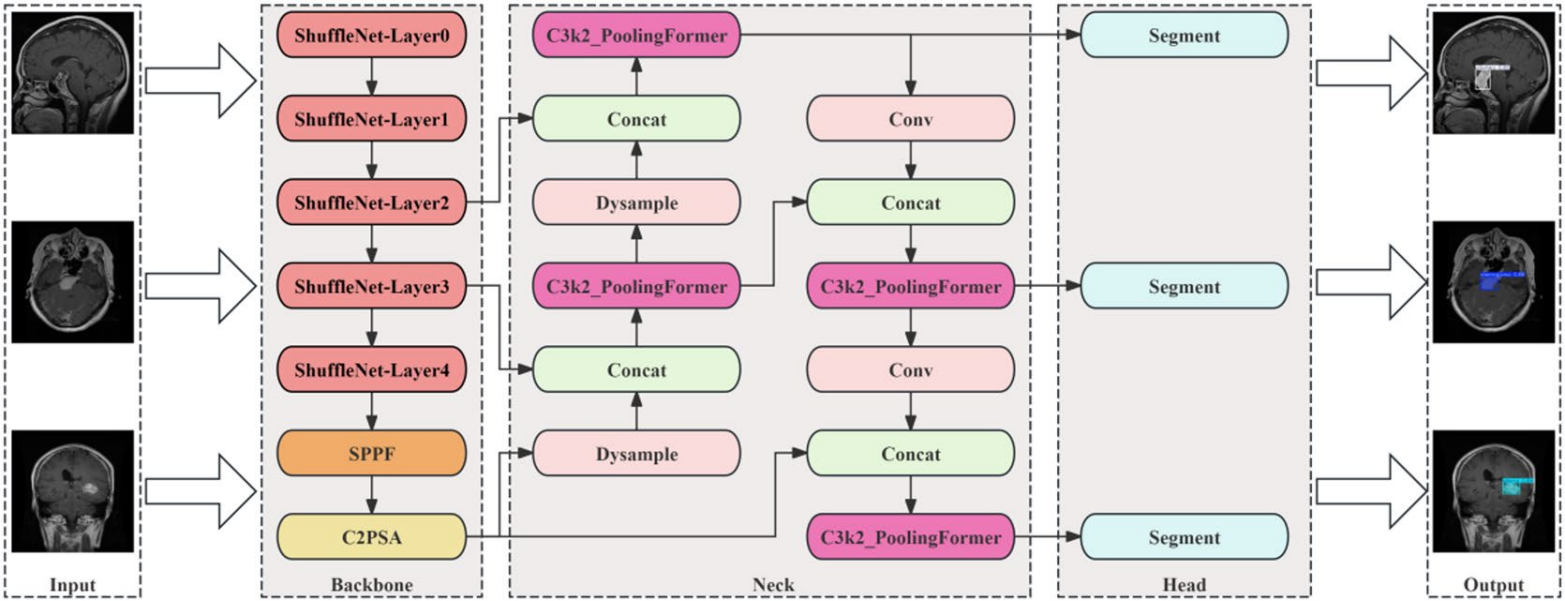
Improved YOLO11n-seg network structure.

Although YOLO11 has achieved significant improvements in precision and lightweighting for brain tumor MRI image recognition and segmentation tasks, repeated convolution and pooling operations in its architecture may lead to dilution or loss of fine-grained features in small-volume tumors during feature map compression. Additionally, the high heterogeneity of brain tumors in structure, morphology, edges, and location can induce model bias toward negative predictions, increasing the risk of false negatives. To address these challenges, targeted improvement strategies are proposed in this study to further enhance recognition, detection accuracy, and segmentation precision while maintaining lightweight characteristics. However, directly selecting larger-scale variants would substantially increase parameters and computational complexity, constraining deployment in resource-constrained clinical environments. Therefore, optimizations are performed based on the smallest-scale variant “n” of the YOLO11n-Seg model, with main improvements including: (1) replacing the backbone network with ShuffleNet V1 for lightweight feature extraction; (2) introducing the DySample dynamic upsampling mechanism to enhance detail recovery; (3) improving the C3k2 module to C3k2-PoolingFormer for optimized cross-scale feature fusion. The improved network structure is shown in Fig. 2.

### ShuffleNet V1 lightweight network

Extending YOLO series models to instance segmentation has achieved excellent balance, but the default backbone still suffers from large parameter counts and high computational complexity, limiting deployment on embedded devices. To overcome this bottleneck, an efficient lightweight architecture is adopted: ShuffleNet v1 replaces the original YOLO11 backbone to construct an instance segmentation network that balances high speed and high precision.

ShuffleNet v1, proposed by the Megvii Technology team, is an advanced CNN architecture with extremely high computational efficiency^28^. Its core concepts involve introducing two novel operations—pointwise grouped convolution and channel shuffle—to significantly reduce computational complexity while maintaining model precision. (1) Pointwise grouped convolution reduces the computational complexity of 1 × 1 convolutions by applying grouped convolutions at the 1 × 1 layer, ensuring each convolution operates only on corresponding input channel groups, thereby substantially lowering costs; (2) Channel shuffle addresses the lack of inter-group information interaction in group convolutions by uniformly shuffling channel information in input feature maps. Specifically, for a convolutional layer with g groups and g×n output channels, the output channel dimension is reshaped to (g, n), transposed, and then flattened as input for the next layer. This operation ensures input and output channels are fully correlated.

Assume an input feature map with width Wi, height Hi, channel count Ci, output channels Co, and kernel size K×K. Parameter counts for each convolution type are calculated as follows: (1) standard convolution (sconv) parameters: P_sc_=C_i_×C_o_×K×K; (2) group convolution (gc) parameters: P_gc_=×C_o_×K×K; (3) depthwise separable convolution (dw) parameters: P_dw_ = C_i_×K×K. Thus, standard convolution has the highest parameter count, group convolution is 1/g of that, and depthwise separable convolution has the lowest, at 1/C_o_ of standard. Combining group and depthwise separable convolutions greatly reduces network parameters and computations.

**Fig. 3:**
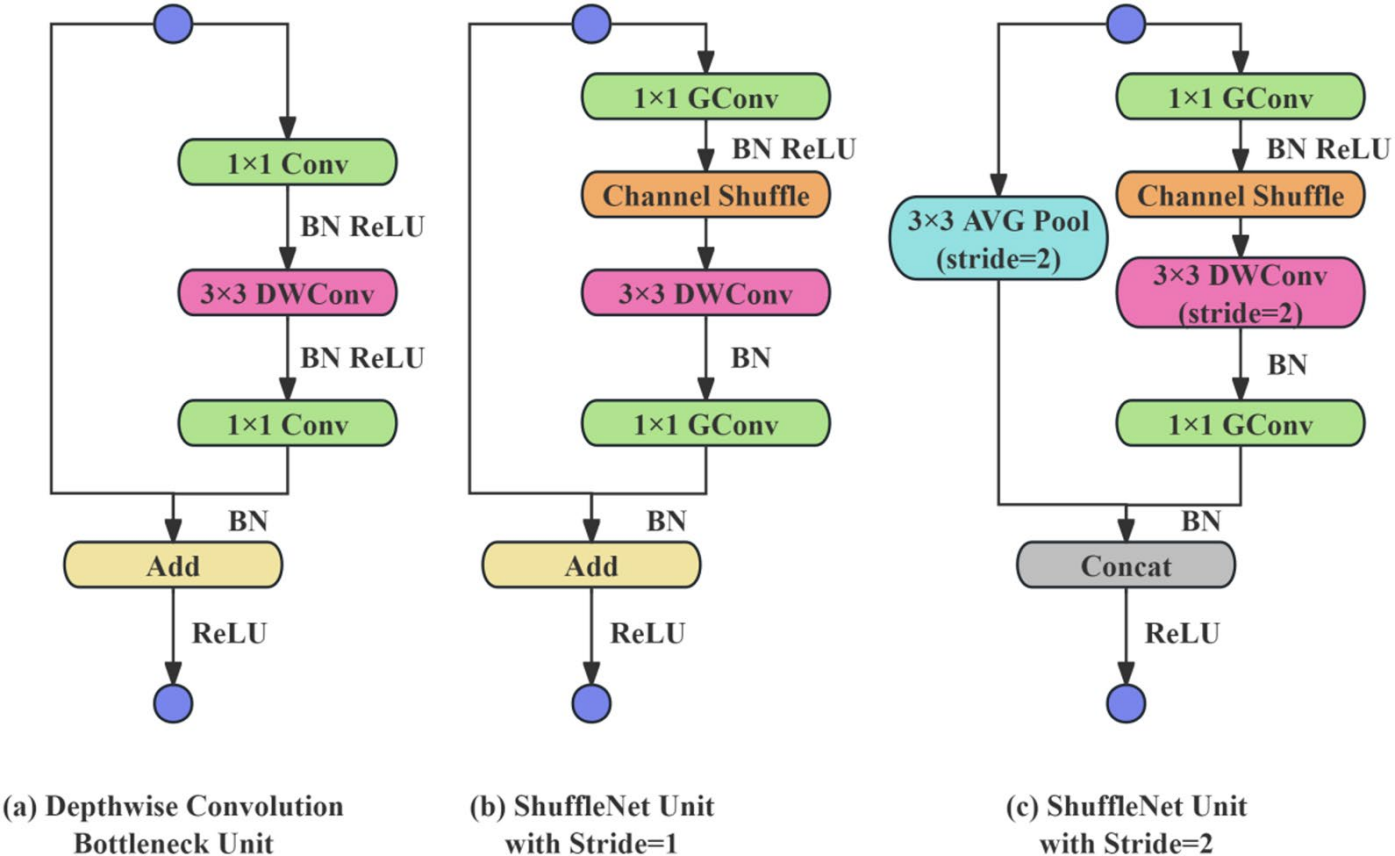
Basic module of ShuffleNet v1.

The ShuffleNet unit is designed based on bottleneck unit principles. In its residual branch, a computationally economical 3 × 3 depthwise separable convolution is applied to the 3 × 3 layer. The first 1 × 1 layer is replaced with pointwise grouped convolution followed by channel shuffle, and the second pointwise grouped convolution restores channel dimensions to match the shortcut path. When stride is applied, a 3 × 3 average pooling is added to the shortcut path, and element-wise addition is replaced with channel concatenation. ShuffleNet V1 is composed of stacked ShuffleNet units divided into three stages, with the first building block in each stage applying stride = 2, maintaining other hyperparameters within the stage, and doubling output channels for the next stage. Network connectivity sparsity and complexity are controlled by adjusting group number g and channel scaling factor s to achieve lightweight feature extraction. The basic module network structure of ShuffleNet V1 is shown in Fig. 3.

### Introduction of DySample upsampling

**Fig. 4:**
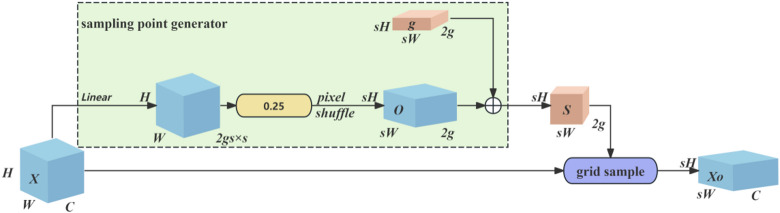
DySample network structure.

YOLO11 employs traditional nearest-neighbor interpolation for upsampling, which may fail to effectively capture feature details and semantic information, impacting model performance in prediction tasks. DySample is a lightweight and efficient upsampling algorithm that uses content-aware dynamic upsampling to adjust sampling positions based on local image features, more accurately restoring feature point positions and details^29^. The DySample upsampling process consists primarily of generating sampling point set S and dynamic feature resampling, as shown in Fig. 4.

Its point sampling concept efficiently conserves computational resources. The sampling point generation process is as follows: (1) Based on input feature map size and upsampling factor s, a regular grid coordinate G representing initial sampling positions is generated. (2) Dynamic offsets O are generated. Input feature map X first passes through a linear convolutional layer to produce initial offsets, constrained by a static range factor (0.25), then undergoes pixel rearrangement to redistribute channel information to high-resolution spatial dimensions, yielding offset set O. (3) Dynamic offsets O are added to regular grid sampling coordinates G to obtain the final dynamic sampling point set S.

The dynamic feature resampling process uses PyTorch’s built-in grid_sample function with bilinear interpolation on input feature map X based on dynamic sampling point set S to smoothly generate high-resolution feature values, resulting in high-resolution feature map X′.

**Fig. 5:**
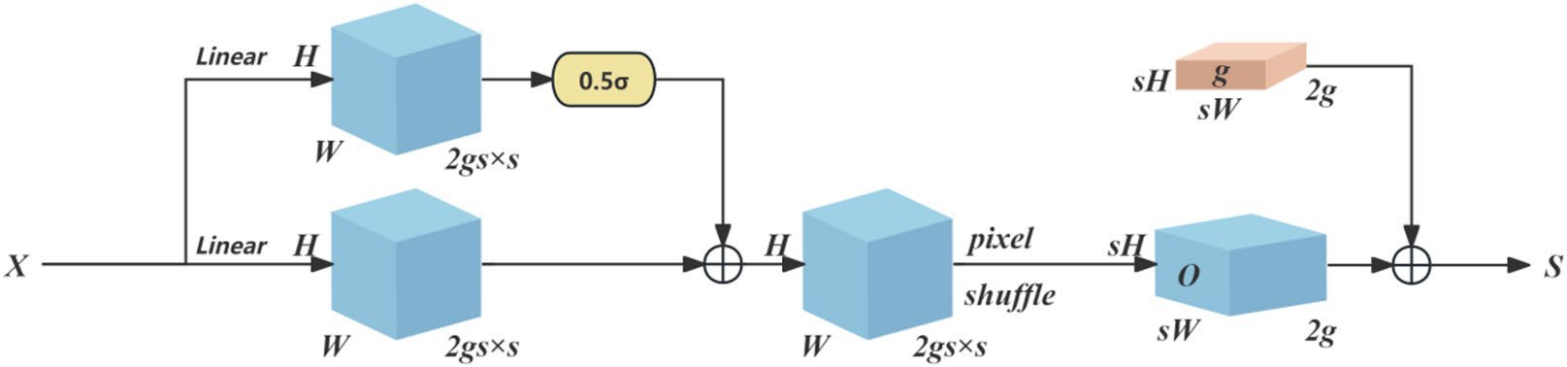
Dynamic range factor.

To increase offset flexibility, dynamic per-point range factors are generated via linear projection of input features, multiplied point-wise by a sigmoid function and 0.5 static range factor to ensure equivalence with the original static constraint, as shown in Fig. 5.

### Improved C3k2 module

In the YOLO11 segmentation framework adopted in this study, the C3k2 module serves as a core component in the neck network, providing multi-scale feature extraction and fusion. It is an enhanced version of the C3 module from YOLOv7 and YOLOv8, designed to optimize feature fusion and computational efficiency through Cross Stage Partial (CSP) structures. The C3k2 module typically includes a backbone path and multiple bottleneck branches, where the backbone path uses convolutional layers for downsampling and channel adjustment, branches extract multi-scale features via repeated bottleneck blocks, and features are fused via concatenation. This design replaces the previous C2f block in YOLO11, further improving speed and accuracy, particularly in real-time object detection tasks. However, in medical imaging tasks like brain tumor segmentation, the C3k2 module has potential limitations: reliance on traditional convolutions may lead to insufficient capture of complex tumor boundaries and fine-grained textures, while computational overhead may be high for high-resolution MRI images, posing challenges for deployment in resource-limited scenarios. Thus, efficient lightweight improvements to the C3k2 module in YOLO11 are urgently needed.

**Fig. 6:**
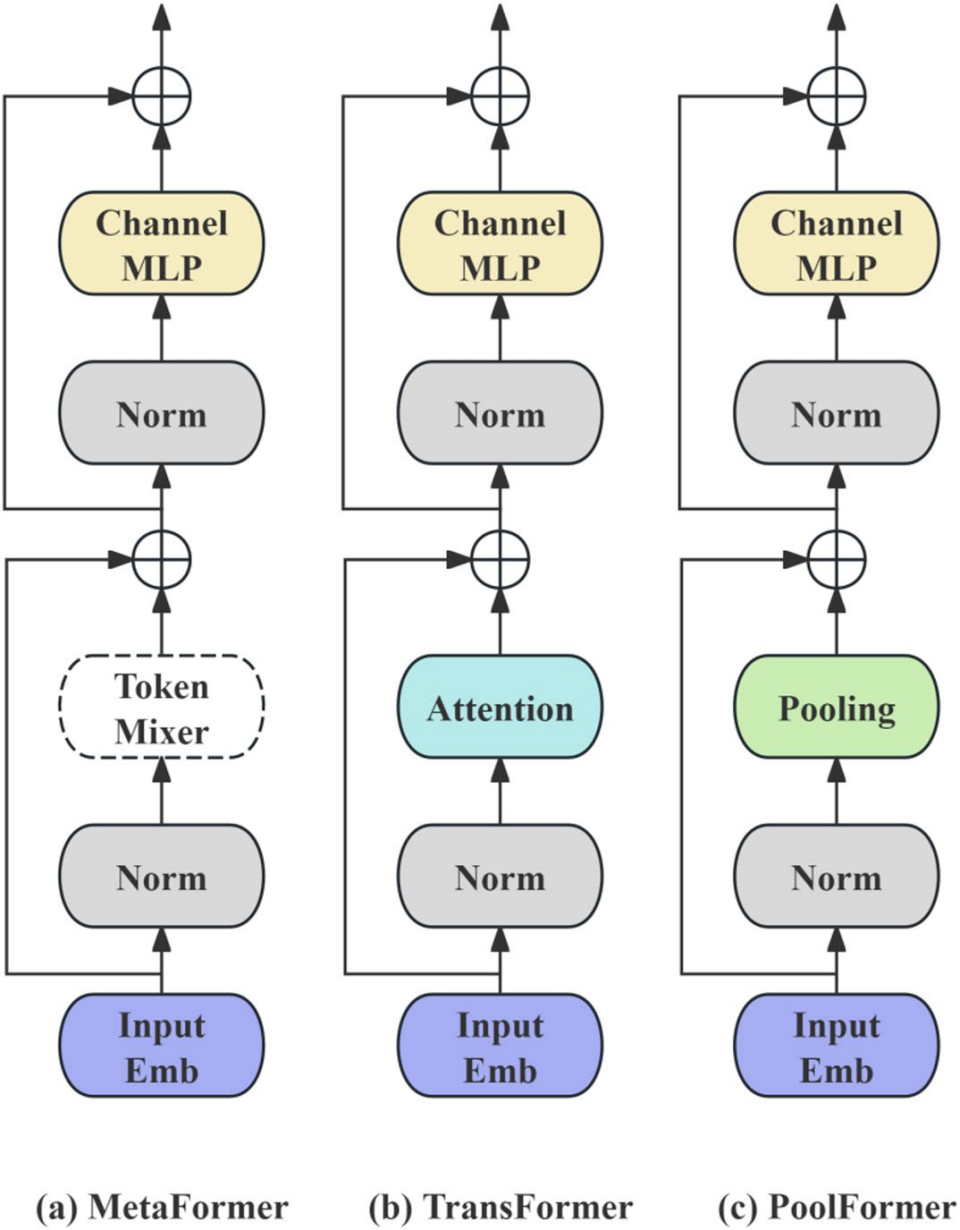
PoolFormer network architecture comparison.

**Fig. 7:**
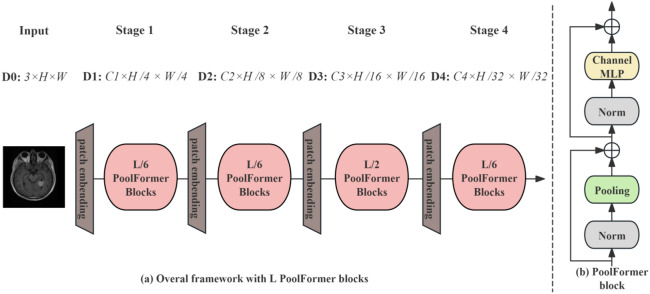
PoolFormer hierarchical architecture.

PoolFormer is an efficient visual Transformer variant proposed by Sea AI Lab in 2022^30^, as shown in Fig. 6. Based on the MetaFormer concept, it replaces the computationally intensive self-attention mechanism in Transformer architectures with simple spatial pooling as the Token Mixer module, significantly reducing computational complexity and parameters while maintaining competitive performance in tasks such as image classification, object detection, and semantic segmentation. The overall PoolFormer architecture uses a hierarchical design, typically divided into 4 stages, each containing multiple PoolFormer blocks, as shown in Fig. 7. Each PoolFormer block’s core structure includes: (1) Patch Embedding: In the first stage, input images are converted to patch tokens via convolution; (2) Token Mixer: Average pooling is used for local feature aggregation instead of global attention, focusing more on local spatial relationships and reducing FLOPs; (3) Channel Mixer: Multi-layer perceptron (MLP) layers perform channel-dimensional feature transformations, typically including Layer Normalization, GELU activation, and DropPath regularization; (4) Residual Connections: Each block uses residual connections for training stability. The main computational processes are as follows:

1 $$\begin{array}{*{20}c} {{\text{X = InputEmb(L)}}} \\ \end{array}$$

2 $$\begin{array}{*{20}c} {{\text{Y = pool(Norm(X)) + X}}} \\ \end{array}$$

3 $$\begin{array}{*{20}c} {Z~ = ~\sigma (Norm(Y)W_{1} )W_{2} ~ + ~Y} \\ \end{array}$$

Where L is input data; Pool denotes average pooling; Norm denotes group normalization; $\:{\mathrm{W}}_{1}$ and $\:{\mathrm{W}}_{2}$ are learnable parameters in MLP; σ is the GELU activation function.

**Fig. 8:**
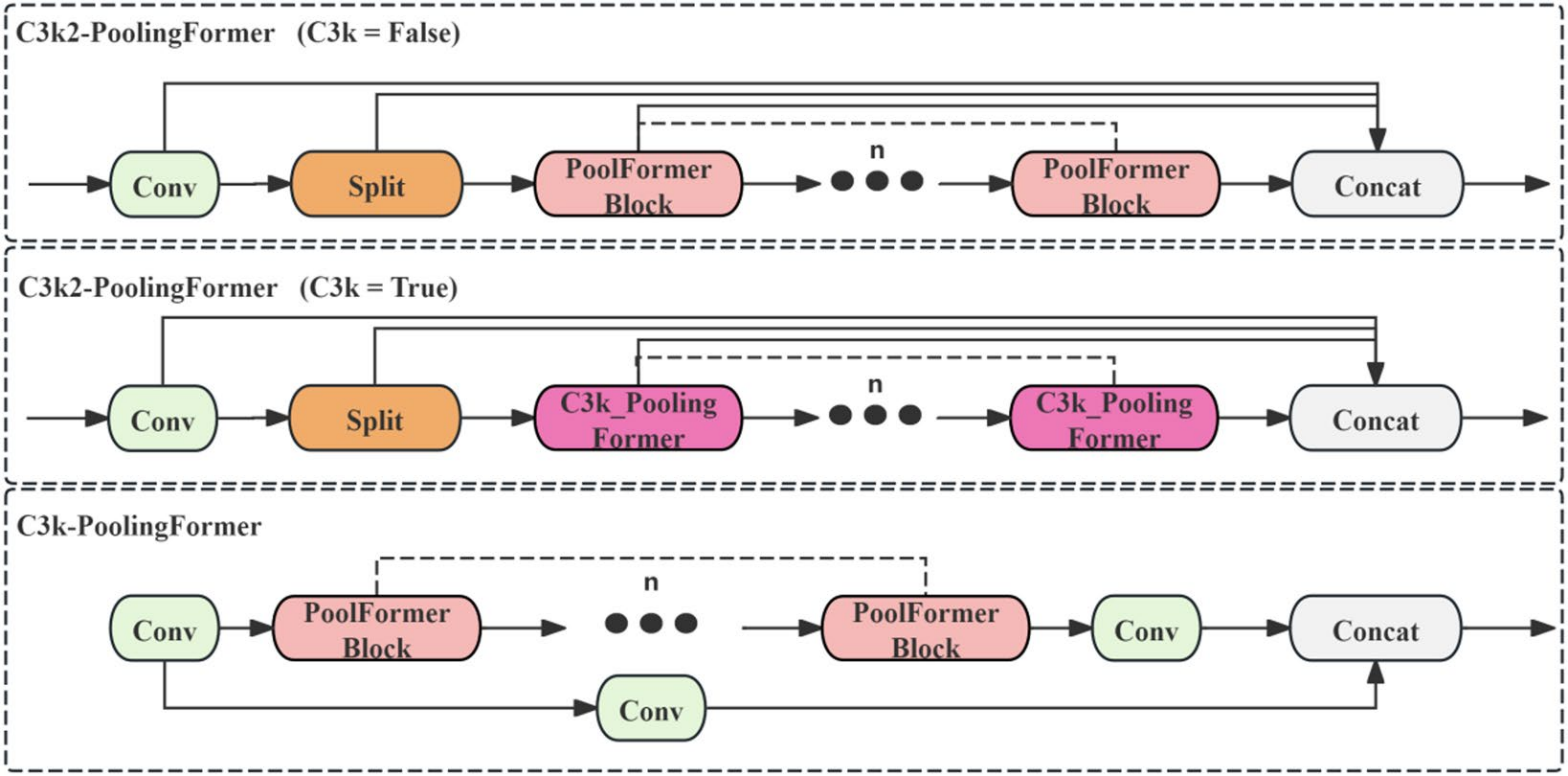
C3k2-PoolingFormer network architecture.

Based on this, the C3k2-PoolingFormer module is proposed to improve the original C3k2 module in YOLO11. Specifically, this module retains the CSP structural framework of C3k2 but replaces bottleneck blocks in branch paths with PoolFormer blocks. The C3k2-PoolingFormer module dynamically configures its processing paths via built-in parameters to address varying task demands. When the parameter is “True,” the C3k-PoolingFormer path is activated, with its core advantage lying in utilizing PoolFormer for efficient token interaction, significantly enhancing local feature representation capabilities, excelling in handling brain tumor features in complex MRI backgrounds. When the parameter is “False,” the module switches to the PoolFormer path, which enhances global context awareness on a lightweight basis, capturing long-range dependencies crucial for understanding image spatial relationships. The network architecture is shown in Fig. 8.

### Data acquisition

Medical imaging data used in this study were sourced from two distinct open public datasets. The first dataset is the Figshare brain tumor dataset provided by Guangzhou Campus of Southern Medical University^31,32^, containing 3064 MRI images from 233 patients, covering three main brain tumor types: meningioma (*n* = 708), glioma (*n* = 1426), and pituitary adenoma (*n* = 930). This dataset, named Dataset 1, was used for model training and internal testing to evaluate performance. The second dataset is from the Kaggle platform^33^, with 300 randomly selected MRI images (100 each of meningioma, glioma, and pituitary adenoma) serving as an external test set for model performance evaluation.

**Fig. 9:**
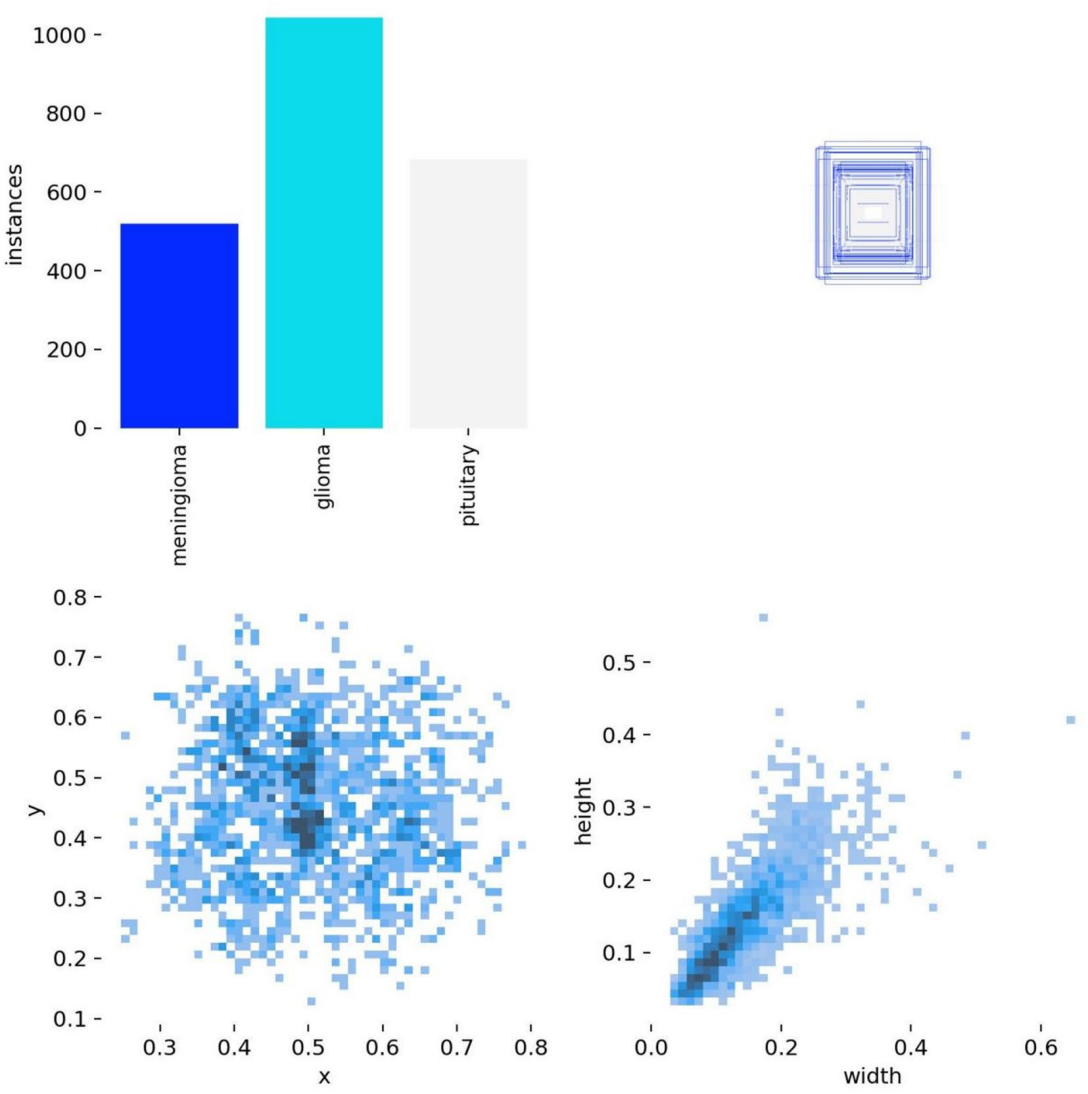
Annotation status of the training set.

**Fig. 10:**
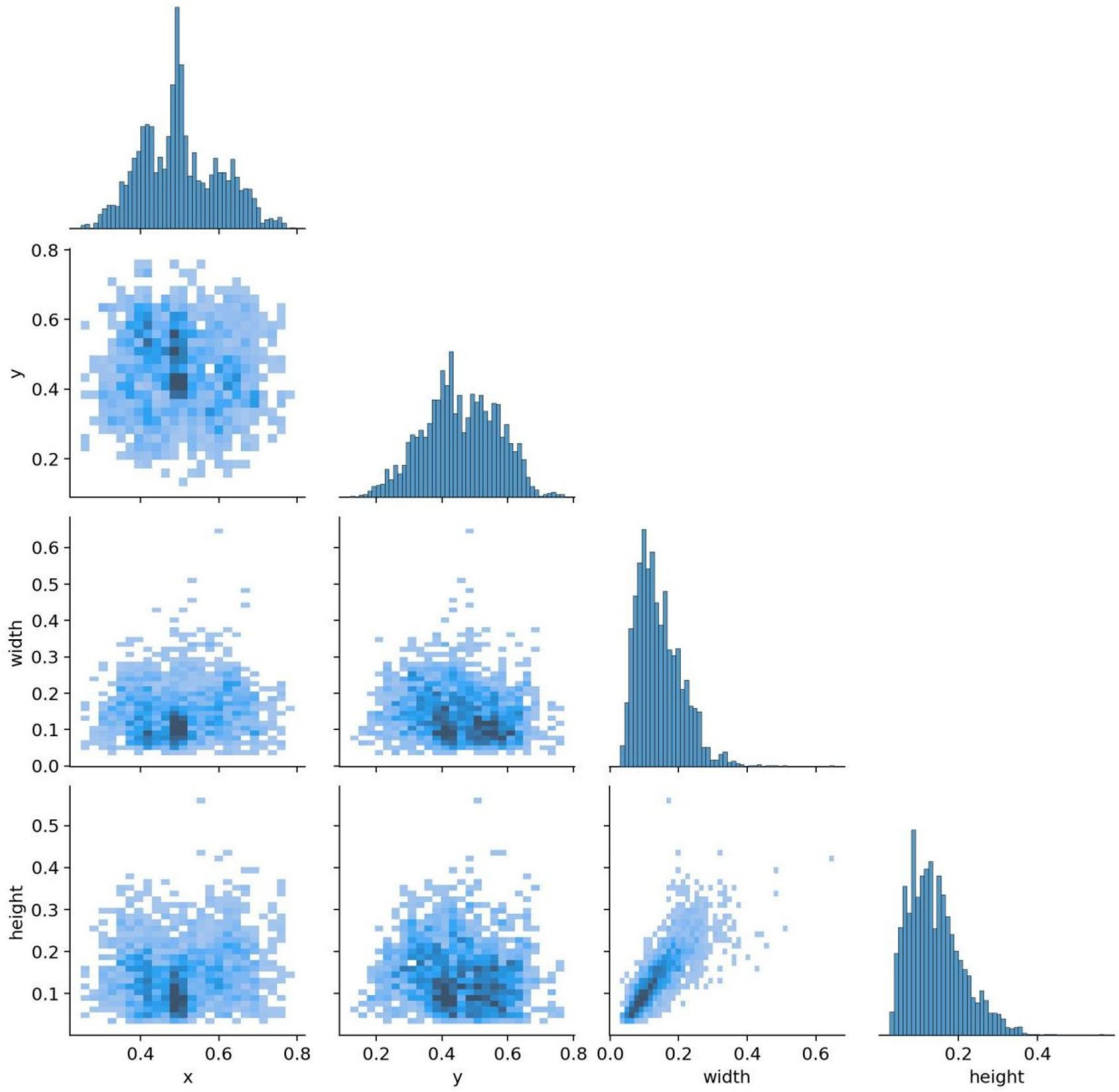
Annotation correlation map of the training set.

All images were pre-annotated for segmentation targets using LabelImg by two radiologists with intermediate titles or above, with results reviewed on-site by two radiologists with associate senior titles or above to ensure experimental accuracy. Five-fold cross-validation was employed for model training in this study. From Dataset 1, 2800 images were randomly divided into 5 equal subsets of 560 images each. These were split in an 8:2 ratio into training and validation sets, with 4 subsets as training and the remaining as validation, cycled five times to create five different data distribution combinations. The remaining 264 images served as an internal independent test set to evaluate the optimal model’s segmentation performance. The training set was used for parameter learning; the validation set independently monitored the training process and triggered early stopping; the test set was strictly isolated for final performance evaluation only. This strategy detects overfitting tendencies in real time during training, ensuring optimal model performance. Annotation distribution characteristics and inter-class correlations in the training dataset were visualized through distribution statistics in Figs. 9 and 10.

### Experimental environment configuration

Experiments were conducted on Windows 11 operating system, with CPU model Intel(R) Core(TM) Ultra 9 275 HX, GPU model NVIDIA GeForce GTX 5070 Laptop, 16G RAM, deep learning framework PyTorch-2.8.0, CUDA-12.9, and Python-3.9.23. Model hyperparameters are shown in Table 1.

**Table 1:** Model hyperparameter settings.

Optimizer	Initial learning rate	Batch size	Epochs	IoU	Momentum	Weight decay
AdamW	0.01	16	200	0.7	0.937	0.0005

Image size	Final learning rate	Mosaic	Close mosaic	Warmup epochs	Conf threshold	Cls loss gain
640 × 640	0.01	1.0	10	3.0	0.25	0.5

### Experimental evaluation metrics

Multiple evaluation metrics were adopted in this study to comprehensively assess model performance in brain tumor MRI image recognition and segmentation tasks. These metrics include precision (Precision), recall (Recall), mean average precision (mAP), intersection over union (IoU), Dice Coefficient (Dice), and 95% Hausdorff Distance (HD95), along with model computational complexity indicators such as GFLOPs (Giga Floating Point Operations). Here, mAP50 denotes average precision at IoU = 0.5, while mAP50-95 represents average precision across IoU from 0.5 to 0.95, providing a stricter and more comprehensive evaluation. These metrics are particularly suitable for medical image processing, quantifying model accuracy and robustness in detecting brain tumor positions, classifying tumor types, and precisely segmenting tumor boundaries, while assessing computational efficiency and lightweighting for clinical deployment. Calculation formulas for evaluation metrics are shown in Table 2.

**Table 2:** Experimental evaluation metrics.

Indicator	Calculation formula
Precision	$\:\mathbf{P}=\frac{\mathbf{T}\mathbf{P}}{\mathbf{T}\mathbf{P}+\mathbf{F}\mathbf{P}}$
Recall	$\:\mathbf{R}=\frac{\mathbf{T}\mathbf{P}}{\mathbf{T}\mathbf{P}+\mathbf{F}\mathbf{N}}$
mean Average Precision (mAP)	$\:\mathbf{m}\mathbf{A}\mathbf{P}=\frac{1}{\mathbf{N}}\sum\:_{\mathbf{i}=1}^{\mathbf{N}}\mathbf{A}{\mathbf{P}}_{\mathbf{i}}$
Intersection over Union (IoU)	$\:\mathbf{I}\mathbf{o}\mathbf{U}=\frac{\mathbf{T}\mathbf{P}}{\mathbf{T}\mathbf{P}+\mathbf{F}\mathbf{P}+\mathbf{F}\mathbf{N}}$
Mean Intersection over Union (mIoU)	$\:\mathbf{m}\mathbf{I}\mathbf{o}\mathbf{U}=\frac{1}{\mathbf{N}}\sum\:_{\mathbf{i}=1}^{\mathbf{N}}\mathbf{I}\mathbf{o}{\mathbf{U}}_{\mathbf{i}}$
Dice coefficient	$\:\mathbf{D}\mathbf{i}\mathbf{c}\mathbf{e}=\frac{2\times\:\mathbf{T}\mathbf{P}}{2\times\:\mathbf{T}\mathbf{P}+\mathbf{F}\mathbf{P}+\mathbf{F}\mathbf{N}}$
95% Hausdorff Distance (HD_95_)	$\:{\mathrm{d}}_{\mathrm{Hausdorff}}\mathrm{(}\mathrm{X}\mathrm{,}\mathrm{Y}\mathrm{)=}\mathrm{max}\left\{\underset{\mathrm{x}\in\mathrm{X}}{\mathrm{max}}\underset{\mathrm{y}\in\mathrm{Y}}{\mathrm{min}}\mathrm{d}\mathrm{(}\mathrm{x}\mathrm{,}\mathrm{y}\mathrm{),\:}\underset{\mathrm{y}\in\mathrm{Y}}{\mathrm{max}}\underset{\mathrm{x}\in\mathrm{X}}{\mathrm{min}}\mathrm{d}\mathrm{(}\mathrm{x}\mathrm{,}\mathrm{y}\mathrm{)}\right\}$

In the formulas, TP represents true positives, FP represents false positives, FN represents false negatives, and N is the number of classes. *X* and *Y* represent the sets of boundary points for the prediction and ground truth, respectively, and *d* denotes the Euclidean distance between points. mAP50 was selected as the primary evaluation metric in this experiment to verify the proposed model’s effectiveness in brain tumor MRI image recognition and segmentation tasks, thereby comprehensively assessing the method’s practicality and computational efficiency. Additionally, to further analyze model reliability in clinical applications, mean IoU (mIoU) and Dice coefficient (Dice) were calculated as supplementary segmentation performance metrics, and HD_95_ was introduced to evaluate the precision of tumor boundary delineation. Finally, GFLOPs were compared to evaluate lightweighting efficiency improvements before and after enhancements, ensuring practicality in resource-limited environments.

## Results and analysis

### Ablation experiments

**Fig. 11:**
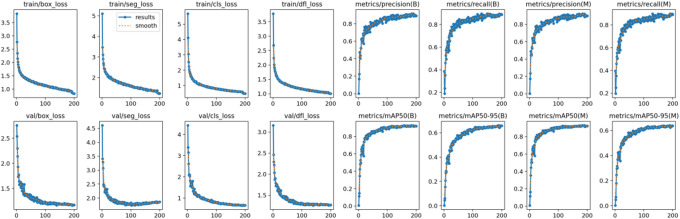
YOLO11n-seg training curve.

**Fig. 12:**
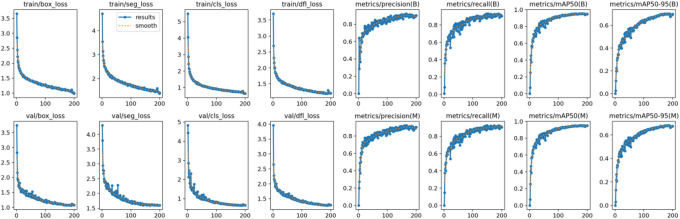
YOLO-LS training curve.

To verify the contributions of the proposed improvement modules to model performance, ablation experiments were conducted based on the YOLO11n-seg model. Experiments used the internal independent test set from Dataset 1 (264 images) for evaluation, with metrics including mAP50, precision (Precision), recall (Recall), mean IoU (mIoU), Dice coefficient (Dice), 95% Hausdorff Distance (HD_95_) and GFLOPs. All models were trained under identical experimental environments, hyperparameter settings, and five-fold cross-validation to ensure result robustness. All performance metrics are reported as mean ± standard deviation across the five folds. Paired t-tests were conducted to assess the statistical significance of differences between the proposed YOLO-LS and each baseline configuration, with *p* < 0.05 considered statistically significant. Ablation experiments progressively introduced three main improvements: (1) replacing the backbone with ShuffleNet V1; (2) introducing DySample upsampling; (3) improving the C3k2 module to C3k2-PoolingFormer. Training curves for the YOLO11n-seg and YOLO-LS models are shown in Figs. 11 and 12.

**Table 3:** Ablation experiment results.

ShuffleNetV1	Dysample	C3k2-PoolingFormer	P(B)	R(B)	mAP50(B)	P(M)	R(M)	mAP50(M)	mIoU	Dice	HD_95_(mm)	GFLOPs
			0.914 ± 0.013	0.873 ± 0.010	0.924 ± 0.010	0.920 ± 0.010	0.877 ± 0.016	0.929 ± 0.013	0.815 ± 0.017	0.898 ± 0.009	5.03 ± 0.33	9.6
√			0.906 ± 0.008	0.893 ± 0.010	0.934 ± 0.009	0.914 ± 0.008	0.892 ± 0.010	0.937 ± 0.010	0.823 ± 0.014	0.903 ± 0.010	4.68 ± 0.37	8.4
	√		0.916 ± 0.014	0.883 ± 0.008	0.936 ± 0.011	0.922 ± 0.014	0.885 ± 0.011	0.939 ± 0.007	0.823 ± 0.018	0.903 ± 0.011	4.68 ± 0.24	9.5
		√	0.913 ± 0.009	0.880 ± 0.015	0.931 ± 0.010	0.920 ± 0.014	0.881 ± 0.014	0.933 ± 0.009	0.818 ± 0.018	0.900 ± 0.011	4.79 ± 0.36	9.2
√	√		0.908 ± 0.015	0.903 ± 0.013	0.946 ± 0.011	0.916 ± 0.013	0.900 ± 0.014	0.947 ± 0.006	0.831 ± 0.012	0.908 ± 0.010	4.41 ± 0.20	8.3
√		√	0.905 ± 0.010	0.900 ± 0.009	0.941 ± 0.008	0.914 ± 0.013	0.896 ± 0.011	0.941 ± 0.008	0.826 ± 0.012	0.905 ± 0.010	4.56 ± 0.46	8.2
	√	√	0.915 ± 0.013	0.890 ± 0.013	0.943 ± 0.007	0.922 ± 0.014	0.889 ± 0.009	0.943 ± 0.008	0.827 ± 0.018	0.905 ± 0.013	4.38 ± 0.29	8.9
√	√	√	0.907 ± 0.013	0.910 ± 0.015	0.953 ± 0.011	0.916 ± 0.010	0.904 ± 0.008	0.951 ± 0.008	0.835 ± 0.012	0.910 ± 0.010	4.35 ± 0.37	8.1

Values are reported as mean ± standard deviation across five-fold cross-validation. Statistical significance was assessed using paired t-tests between the full YOLO-LS model and the baseline YOLO11n-seg across five folds. Key comparisons: mAP50(B) *p* = 0.003; Dice *p* = 0.038; HD_95_ *p* = 0.006. GFLOPs is a deterministic architectural metric and does not vary across folds.

**Fig. 13:**
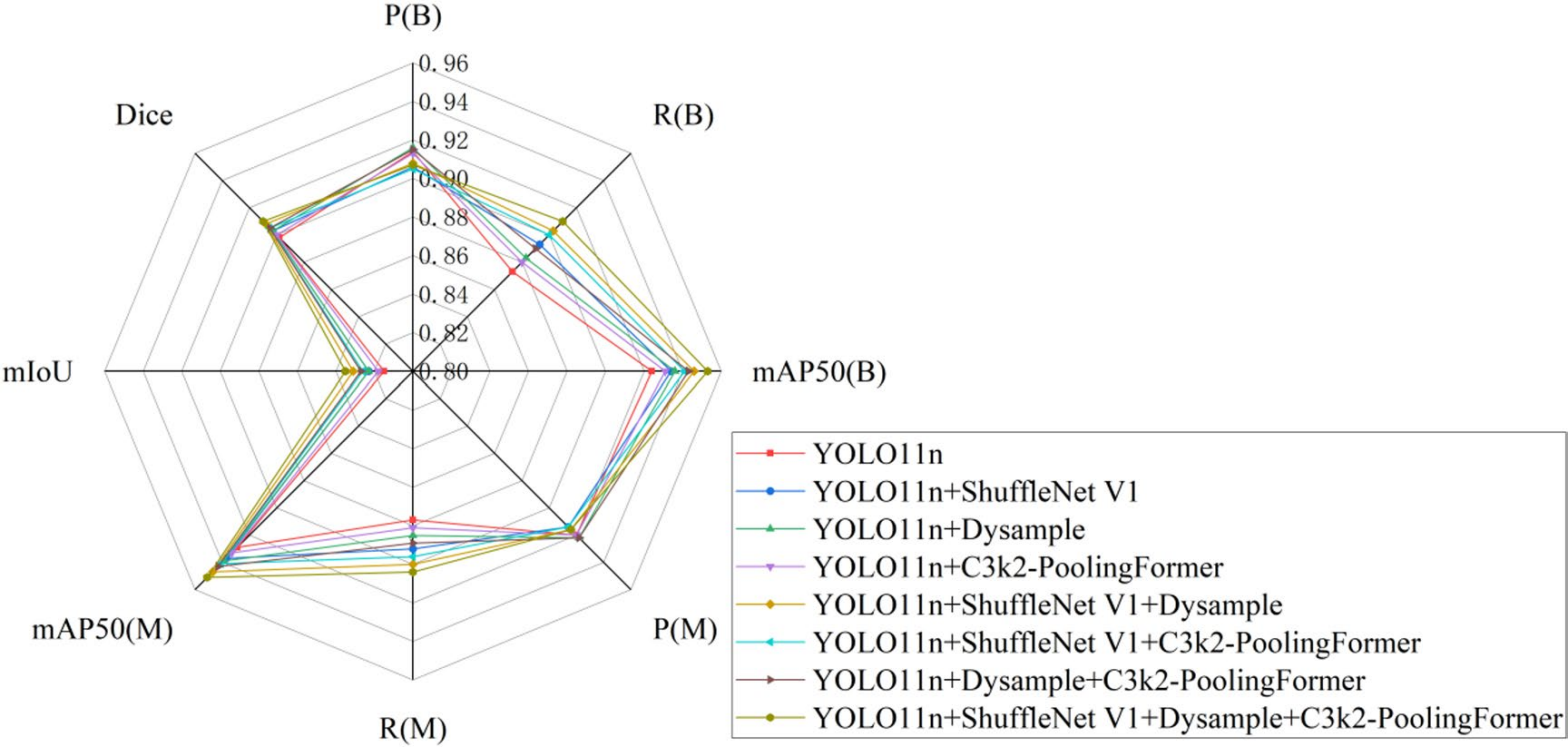
Ablation experiment comparison.

The ablation study results, presented in Table 3; Fig. 13, confirm that the progressive integration of the three proposed modules significantly enhanced the performance of the YOLO11n-seg model in brain tumor MRI segmentation. The baseline YOLO11n-seg model established a solid foundation with a bounding box mAP50 (mAP50(B)) of 0.924 ± 0.010, Precision(B) of 0.914 ± 0.012, Recall(B) of 0.873 ± 0.010, mIoU of 0.815 ± 0.017, and a Dice score of 0.898 ± 0.009. However, with a 95% Hausdorff Distance (HD95) of 5.03 ± 0.31 mm, the baseline exhibited limitations in defining precise boundaries for small or morphologically complex tumors. The introduction of the ShuffleNet V1 backbone addressed computational redundancy; while Precision(B) saw a marginal decline to 0.906, the model achieved a 12.5% reduction in GFLOPs (to 8.4) and an improved Recall(B) of 0.893. Notably, this lightweight architecture enhanced boundary adherence, reducing HD95 to 4.68 mm. Similarly, the standalone application of the DySample dynamic upsampling mechanism raised mAP50(B) to 0.936 and achieved an HD95 of 4.68 mm, indicating that its content-aware sampling effectively preserves fine-grained details in small lesions. Implementing the C3k2-PoolingFormer module alone also yielded gains, achieving an mAP50(B) of 0.931 and an HD95 of 4.79 mm, validating the utility of pooling operations for capturing complex texture features. The integration of all three improvements (YOLO-LS) yielded the optimal configuration: mAP50(B) peaked at 0.953 ± 0.011, Recall(B) at 0.910 ± 0.010, mIoU at 0.835 ± 0.012, and Dice at 0.910 ± 0.010. Most significantly, the model achieved the lowest boundary error with an HD95 of 4.35 ± 0.34 mm, representing a substantial improvement in contour precision compared to the baseline. Paired t-tests across the five folds confirmed that the improvements in mAP50(B) (*p* = 0.003), HD95 (*p* = 0.006), and Dice (*p* = 0.038) were statistically significant relative to the baseline. Simultaneously, computational cost was reduced to 8.1 GFLOPs—a 15.6% decrease. Overall, the proposed improvements increased mAP50(B) by 2.9% points and Dice by 1.2% points while significantly sharpening tumor boundaries. The training curves further demonstrate faster convergence and superior robustness, confirming that YOLO-LS achieves an ideal balance between segmentation precision and computational efficiency, demonstrating significant potential for applications in resource-limited environments.

### YOLO series comparison experiments

To further validate the superiority of the YOLO-LS model in brain tumor MRI image recognition and segmentation tasks, comparison experiments were conducted with other mainstream YOLO series versions, including YOLOv5n-seg, YOLOv8n-seg, YOLO12n-seg, and the baseline YOLO11n-seg model. All comparison models were evaluated on the same internal independent test set from Dataset 1 (264 images), under identical experimental environments, hyperparameter settings, and five-fold cross-validation. All metrics are reported as mean ± standard deviation, and paired t-tests were employed to assess statistical significance. Evaluation metrics aligned with those in ablation experiments. The experiments aimed to examine performance across different YOLO versions in precision, segmentation accuracy, and computational efficiency, particularly for complex tumor morphologies and boundary ambiguity.

**Fig. 14:**
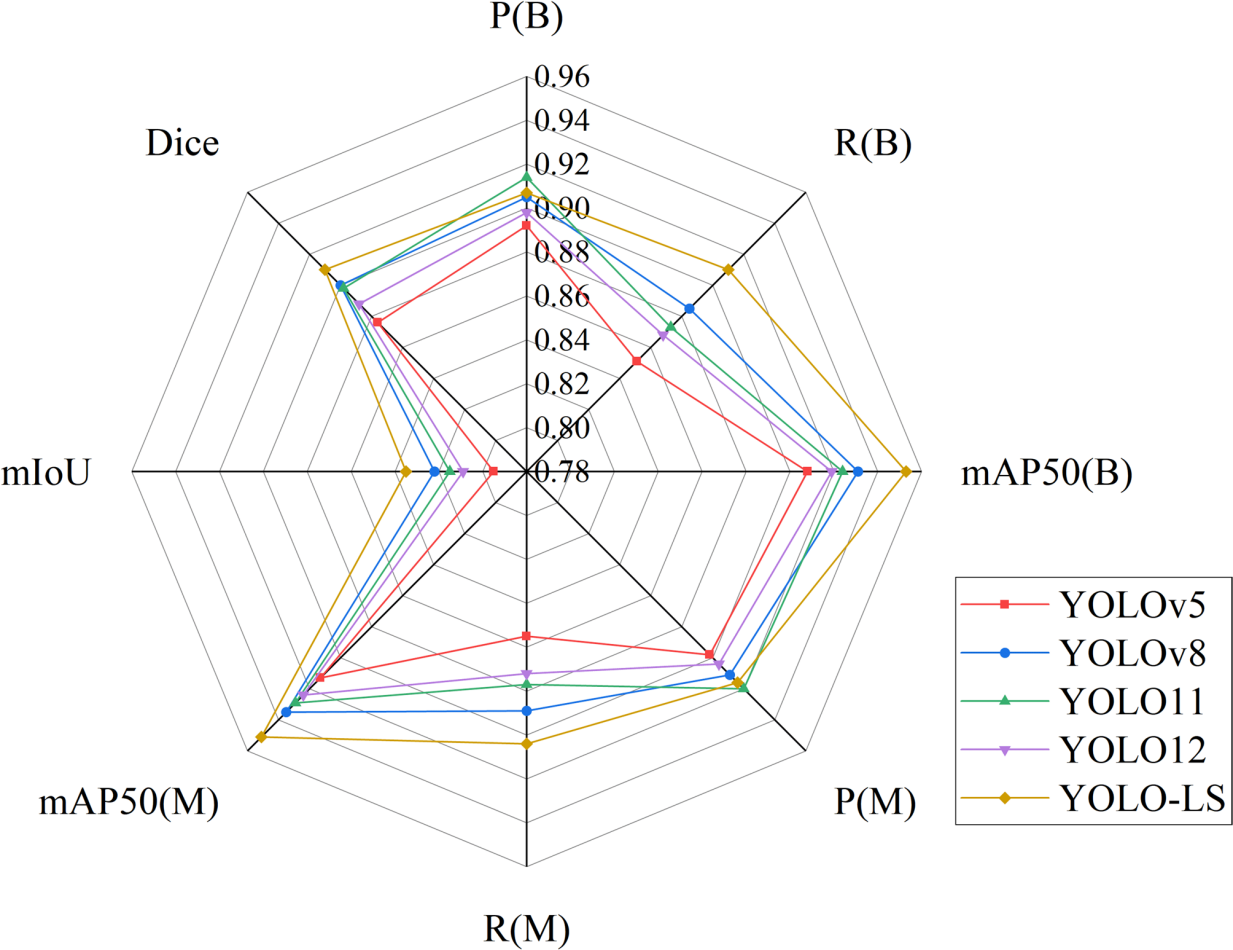
YOLO series experiment comparison.

Comparison results show that the YOLO-LS model outperformed baseline models across multiple metrics. Specifically, the improved model significantly reduced computational complexity while enhancing detection and segmentation precision. This indicates that the improvement strategies effectively addressed feature extraction deficiencies and small-target detection challenges in original YOLO series for medical imaging tasks. Detailed comparison results are shown in Table 4; Fig. 14.

**Table 4:** YOLO series comparison experiment results.

Model	P(B)	R(B)	mAP50(B)	P(M)	R(M)	mAP50(M)	mIoU	Dice	HD_95_(mm)	GFLOPs
YOLOv5n-seg	0.892 ± 0.015	0.851 ± 0.013	0.908 ± 0.013	0.898 ± 0.011	0.855 ± 0.017	0.913 ± 0.012	0.795 ± 0.015	0.876 ± 0.012	6.56 ± 0.44	7.3
YOLOv8n-seg	0.905 ± 0.010	0.885 ± 0.013	0.931 ± 0.011	0.911 ± 0.011	0.889 ± 0.010	0.935 ± 0.012	0.822 ± 0.014	0.900 ± 0.009	4.79 ± 0.23	11.3
YOLO11n-seg	0.914 ± 0.009	0.873 ± 0.015	0.924 ± 0.014	0.920 ± 0.011	0.877 ± 0.011	0.929 ± 0.011	0.815 ± 0.018	0.898 ± 0.012	5.03 ± 0.47	9.6
YOLO12n-seg	0.898 ± 0.015	0.868 ± 0.010	0.919 ± 0.014	0.904 ± 0.013	0.872 ± 0.014	0.924 ± 0.010	0.809 ± 0.015	0.888 ± 0.009	5.45 ± 0.33	9.8
YOLO-LS	0.907 ± 0.012	0.910 ± 0.016	0.953 ± 0.011	0.916 ± 0.010	0.904 ± 0.012	0.951 ± 0.007	0.835 ± 0.017	0.910 ± 0.015	4.35 ± 0.24	8.1

Values are reported as mean ± standard deviation across five-fold cross-validation. Statistical significance was assessed using paired t-tests comparing YOLO-LS against each baseline model. All comparisons of mAP50(B) between YOLO-LS and other models were statistically significant (*p* < 0.05). Specifically: vs. YOLOv5n-seg *p* < 0.001; vs. YOLOv8n-seg *p* = 0.012; vs. YOLO11n-seg *p* = 0.003; vs. YOLO12n-seg *p* = 0.002.

### Comparison experiments with different networks

**Fig. 15:**
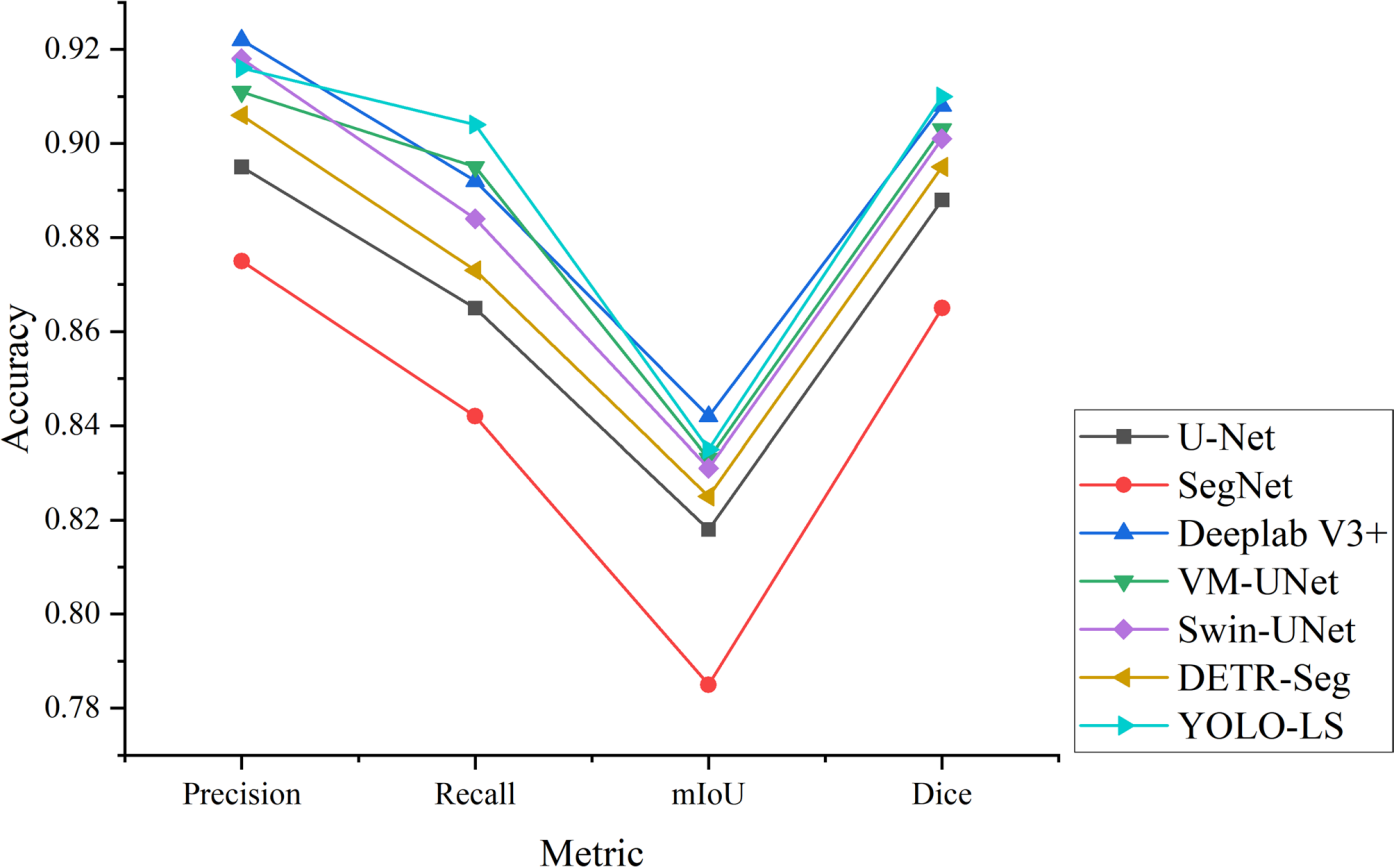
Different network experiment comparison.

To comprehensively evaluate the overall performance of the YOLO-LS model, comparisons were conducted not only with classic CNN-based methods (U-Net, SegNet, Deeplab V3+) but also with recent state-of-the-art (SOTA) architectures representing different mechanisms, including the Transformer-based Swin-UNet, the Mamba-based VM-UNet, and the end-to-end Transformer-based instance segmentation architecture DETR-Seg. To ensure a fair and reproducible comparison, all models were trained and evaluated on the Figshare brain tumor dataset, a widely adopted public benchmark in brain tumor segmentation research^34^, using the same five-fold cross-validation splits, preprocessing pipeline, and hardware environment. All results are reported as mean ± standard deviation, with paired t-tests used for significance testing. The comparison results are presented in Table 5; Fig. 15.

As shown in Table 5; Fig. 15, YOLO-LS achieved the best overall performance among all compared methods. In segmentation quality, YOLO-LS attained the highest mIoU (0.835 ± 0.012) and Dice (0.910 ± 0.010), surpassing all baselines including Deeplab V3+, VM-UNet, Swin-UNet, DETR-Seg, U-Net, and SegNet. Paired t-tests confirmed statistically significant Dice improvements over U-Net (*p* = 0.014), SegNet (*p* < 0.001), and DETR-Seg (*p* = 0.027), while differences with Deeplab V3+ (*p* = 0.478), VM-UNet (*p* = 0.186), and Swin-UNet (*p* = 0.092) were not statistically significant, reflecting the competitive segmentation quality of these architectures. In boundary precision, YOLO-LS achieved the lowest HD₉₅ (4.35 ± 0.28 mm) and the highest Recall (0.904 ± 0.009), indicating superior capability in capturing fine-grained tumor details and minimizing missed detections. Particularly noteworthy is the computational efficiency: YOLO-LS achieved these results at only 8.1 GFLOPs, which is significantly lower than all other methods, demonstrating the effectiveness of the proposed lightweight design in achieving an excellent balance between segmentation accuracy and computational cost for clinical deployment.

**Table 5:** Different network comparison experiment results.

Model	Precision	Recall	mIoU	Dice	HD_95_(mm)	GFLOPs
U-Net	0.895 ± 0.013	0.865 ± 0.015	0.818 ± 0.011	0.888 ± 0.014	5.45 ± 0.36	45.9
SegNet	0.875 ± 0.016	0.842 ± 0.014	0.785 ± 0.012	0.865 ± 0.013	6.82 ± 0.30	32.8
Deeplab V3+	0.922 ± 0.015	0.892 ± 0.015	0.842 ± 0.017	0.908 ± 0.010	4.41 ± 0.19	62.3
VM-UNet	0.911 ± 0.015	0.895 ± 0.016	0.833 ± 0.011	0.903 ± 0.012	4.68 ± 0.24	26.7
Swin-UNet	0.918 ± 0.014	0.884 ± 0.014	0.831 ± 0.011	0.901 ± 0.012	4.75 ± 0.36	48.1
DETR-Seg	0.906 ± 0.010	0.873 ± 0.017	0.825 ± 0.013	0.895 ± 0.010	5.12 ± 0.36	67.4
YOLO-LS	0.916 ± 0.014	0.904 ± 0.010	0.835 ± 0.012	0.910 ± 0.016	4.35 ± 0.31	8.1

Values are reported as mean ± standard deviation across five-fold cross-validation. Statistical significance was assessed using paired t-tests comparing YOLO-LS against each competing architecture. Dice coefficient comparisons: vs. U-Net *p* = 0.014; vs. SegNet *p* < 0.001; vs. Deeplab V3 + *p* = 0.478; vs. VM-UNet *p* = 0.186; vs. Swin-UNet *p* = 0.092; vs. DETR-Seg *p* = 0.027.

**Fig. 16:**
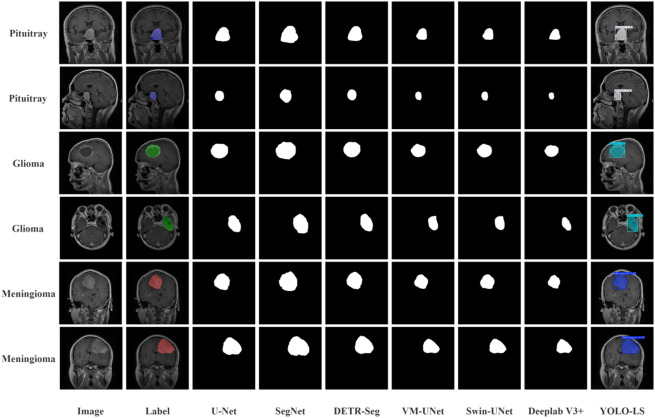
Visual comparison of different network experiments.

To visually corroborate the quantitative findings in Table 5, segmentation effect visualizations were employed for a comprehensive qualitative analysis. As illustrated in Fig. 16, these visualizations provide a direct comparison of how different architectures—ranging from classic CNNs (U-Net, Deeplab V3+) to recent Transformer (Swin-UNet, DETR-Seg) and Mamba (VM-UNet) variants—handle complex tumor morphologies. Specifically, while models like Deeplab V3 + and Swin-UNet effectively identify the tumor bulk, they occasionally exhibit boundary over-smoothing or miss subtle marginal infiltrations. In contrast, YOLO-LS demonstrates superior capability in delineating irregular tumor boundaries and preserving fine-grained heterogeneous textures. This visual evidence strongly aligns with the superior HD95 metric (4.35 mm) reported in Table 5, confirming that the proposed framework not only ensures high detection sensitivity but also delivers the precise boundary definition essential for accurate clinical radiotherapy planning and surgical navigation.

### Generalization capability evaluation

To evaluate the generalization capability of the proposed YOLO-LS model in brain tumor MRI image segmentation tasks, Dataset 2 was used as an external independent test set. This dataset originates from different sources, differing from training Dataset 1 in imaging equipment, patient populations, and image quality, making it suitable for testing model robustness on unseen data. The YOLO-LS model, using the best weights trained on Dataset 1, was directly applied to Dataset 2 for performance evaluation without additional fine-tuning. Evaluation metrics aligned with previous experiments. Specific metrics for each tumor type and overall performance of the YOLO-LS model on the external independent test set are shown in Table 6.

**Table 6:** External test set generalization capability evaluation results.

Category	P(B)	R(B)	mAP50(B)	P(M)	R(M)	mAP50(M)	mIoU	Dice
Meningioma	0.902	0.905	0.938	0.91	0.902	0.936	0.828	0.903
Glioma	0.885	0.888	0.928	0.892	0.885	0.925	0.812	0.888
Pituitary	0.908	0.912	0.942	0.915	0.908	0.94	0.83	0.905
All	0.898	0.902	0.936	0.906	0.898	0.934	0.82	0.895

Based on the evaluation results in Table 6, the YOLO-LS model exhibited excellent overall performance on the external independent test set, with bounding box detection precision (P(B)) of 0.898, recall (R(B)) of 0.902, and mAP50(B) of 0.936; mask segmentation precision (P(M)) of 0.906, recall (R(M)) of 0.898, and mAP50(M) of 0.934. For segmentation quality, mean IoU (mIoU) was 0.82, and Dice coefficient was 0.895. These metrics indicate that the model maintained high levels of recognition, detection, and segmentation performance on unseen data, validating its strong generalization capability.

By tumor type, pituitary adenoma showed the best recognition and segmentation, with mAP50(B) of 0.942 and Dice of 0.905; meningioma followed, with mAP50(B) of 0.938 and Dice of 0.903; glioma metrics were slightly lower but still reached mAP50(B) of 0.928 and Dice of 0.888. This demonstrates the YOLO-LS model’s good adaptability to different pathological types of brain tumors, effectively handling image differences from various data sources. However, glioma showed relatively lower recall and Dice on the external test set, possibly due to its often blurred boundaries and strong heterogeneity in MRI, though overall performance remained acceptable.

**Fig. 17:**
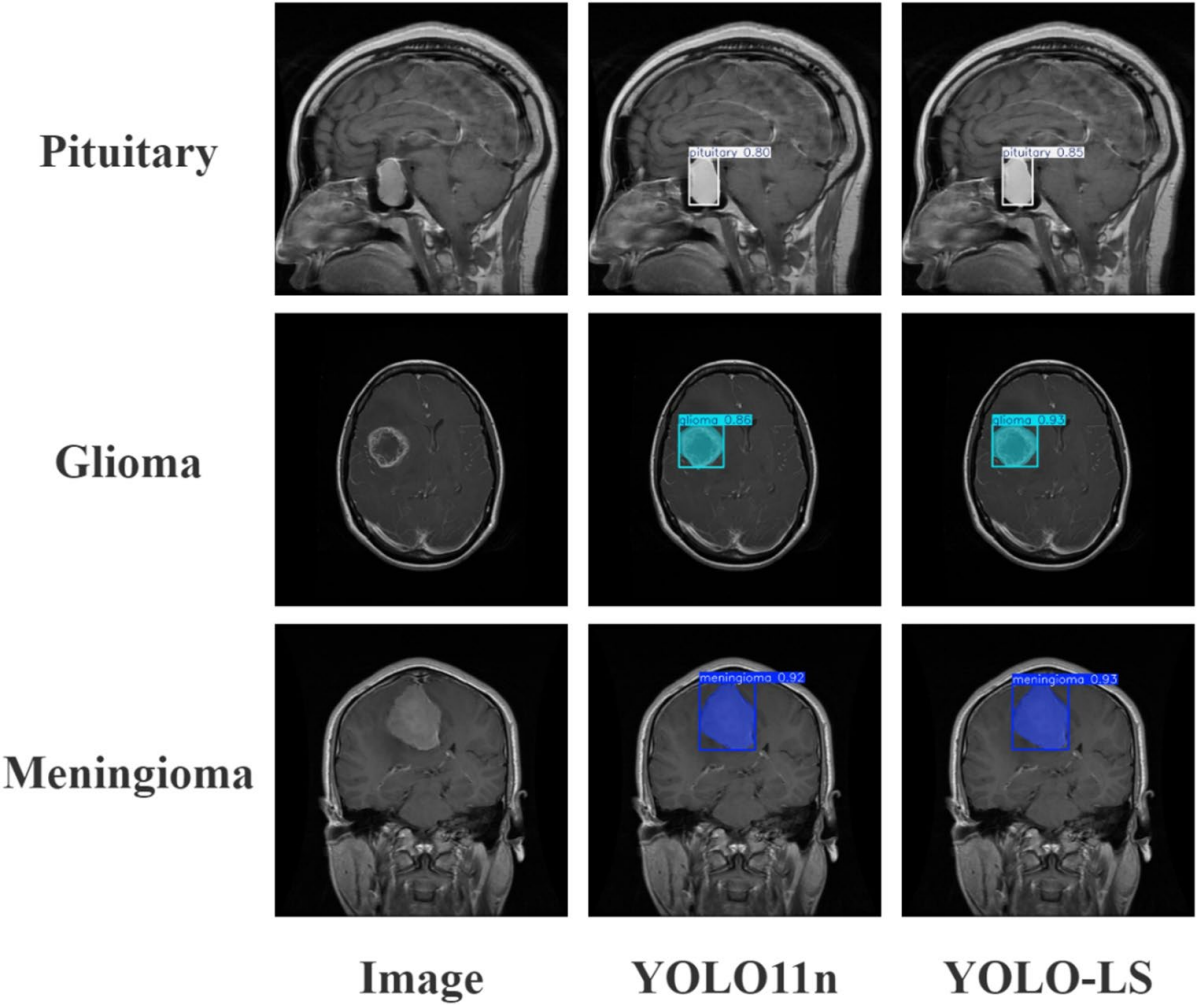
Recognition and segmentation result comparison of models before and after improvements on the external test set.

To more intuitively display the YOLO-LS model’s recognition and segmentation effects on the external test set, visualization results are provided as shown in Fig. 17. This figure compares segmentation outputs of models before and after improvements on typical samples, clearly showing YOLO-LS’s advantages in tumor boundary localization and detail recovery, further validating its potential in practical clinical applications.

### Heatmaps

**Fig. 18:**
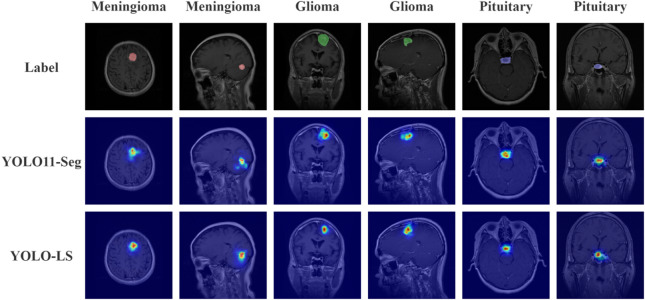
Attention heatmaps for different tumor types.

To deeply analyze the model’s decision-making process and focus on key brain tumor regions, Grad-CAM (Gradient-weighted Class Activation Mapping) was employed to generate heatmaps. This method visualizes the model’s emphasis on different input image regions during prediction by computing gradient weights. Specifically, we targeted the final convolutional layer of the detection head to capture high-level semantic features. The activation maps were computed based on the classification logits of the highest-confidence detection output. This allows us to verify whether the model focuses on the lesion areas driven by tumor-specific features rather than background noise. In the visualizations, red areas indicate high attention, while blue areas indicate low attention. Heatmaps were generated based on YOLO-LS model predictions on MRI images from Dataset 1’s internal test set to verify the model’s correct focus on lesion areas effectively avoiding background noise or irrelevant structures. Heatmap displays for the improved model on MRI images of different brain tumor types are shown in Fig. 18.

Figure 18 heatmap examples (top: label visualization of original images; middle: YOLO11n-Seg model heatmap results; bottom: YOLO-LS model heatmap results after improvements). Results show that YOLO-LS heatmaps more precisely highlight tumor core and edge regions, with more concentrated attention distribution, avoiding dispersion to non-tumor areas. This benefits from the C3k2-PoolingFormer module’s pooling operations enhancing local feature aggregation and DySample upsampling’s detail retention capabilities. For example, in glioma images, heatmaps clearly show model focus on heterogeneous textures and invasive boundaries; in pituitary adenoma images, attention centers on tumor core positions, reducing mis-focus on adjacent normal tissues. Notably, in small tumor cases, improved model activation coverage is higher, further explaining its recall advantages. These visualization results not only validate model interpretability but also provide references for clinicians to understand AI decision bases.

## Discussion

This study presents YOLO-LS, a framework derived from YOLO11n-seg, to enhance brain tumor MRI segmentation efficiency and precision. By integrating three targeted modules, YOLO-LS achieved an mAP50 of 0.953 ± 0.011 with a 15.6% reduction in GFLOPs, demonstrating an effective balance between segmentation quality and lightweight design.

The superior performance is attributed to three targeted optimizations. The ShuffleNet V1 backbone substantially reduced computational complexity via grouped convolutions and channel shuffling, aligning with the demand for lightweight medical image analysis models^35^. The DySample mechanism preserves fine-grained details critical for delineating small, infiltrative lesions, while the C3k2-PoolingFormer module captures long-range context without the computational overhead of standard Transformers.

A notable contribution is the model’s computational efficiency. YOLO-LS achieves a Dice of 0.910 ± 0.010 and HD95 of 4.35 ± 0.34 mm with only 8.1 GFLOPs—substantially lower than U-Net (45.9), Deeplab V3+ (62.3), and other architectures at comparable precision. This highlights the potential for resource-constrained clinical applications, although deployment on embedded platforms would require further hardware-specific validation.

Beyond performance on internal datasets, generalization capability is essential for clinical utility^36,37^. YOLO-LS was validated on an independent external dataset without fine-tuning, achieving a Dice of 0.895 across diverse tumor types, suggesting the model has learned transferable imaging biomarkers rather than overfitting to training data^38,39^. Furthermore, Grad-CAM heatmaps confirm that YOLO-LS focuses more precisely on tumor cores and boundaries compared to the baseline, suppressing irrelevant background noise.

### Limitations and future work

Despite the significant progress achieved, certain limitations persist. First, the training dataset primarily consists of T1-weighted contrast-enhanced MRI (CE-MRI) sequences. While this modality is optimal for visualizing the tumor core, it may limit the precise delineation of peritumoral edema, which is typically better observed in T2-weighted or FLAIR sequences. Second, although generalization was demonstrated, the size of the external independent test set remains relatively small, and ensuring robustness across different institutions, scanners, and imaging protocols remains a broader challenge in the field^40^. Additionally, a slight reduction in recall was observed for gliomas in the external validation compared to other tumor types. This is likely due to the infiltrative nature and blurred boundaries characteristic of gliomas, which present persistent segmentation challenges. Furthermore, while the proposed YOLO-LS demonstrates a substantial reduction in computational cost (8.1 GFLOPs), this study evaluates efficiency solely through GFLOPs under a controlled GPU environment. No deployment or benchmarking has been performed on actual embedded or edge hardware platforms (e.g., NVIDIA Jetson, Intel Neural Compute Stick). The translation from theoretical computational cost to practical inference latency on such platforms depends on multiple hardware-specific factors, including memory bandwidth, operator-level optimization, and model format conversion (e.g., ONNX, TensorRT), which remain to be investigated.

Looking ahead, future investigations will focus on the following directions: (1) Multimodal and Temporal Information Fusion: Research will explore effective strategies to integrate T1, T2, FLAIR, and DWI sequences, alongside Dynamic Contrast-Enhanced MRI (DCE-MRI) or Diffusion Tensor Imaging (DTI), to provide richer imaging biomarkers. (2) Personalized Adaptive Learning: The development of online or meta-learning strategies is planned to enable rapid fine-tuning based on patient-specific follow-up imaging, adapting to morphological changes during treatment. (3) Clinical Workflow Integration: Efforts will be made to interface segmentation results directly with Radiation Therapy Planning Systems (RTPS) or neuronavigation systems to facilitate automatic tumor volume delineation and dose calculation. (4) Prospective Clinical Validation: Rigorous clinical trials are warranted to quantitatively assess the impact of YOLO-LS on diagnostic efficiency and patient outcomes. (5) Collaborative Training with Privacy Protection: To overcome data privacy barriers, Federated Learning techniques will be introduced. This approach will allow for cross-institutional collaborative training without the need to transfer raw data, thereby maximizing the utility of distributed medical resources. (6) Edge-Device Deployment and Benchmarking: Future work will include exporting the YOLO-LS model to optimized inference formats (e.g., ONNX, TensorRT) and benchmarking inference latency, throughput, and power consumption on representative edge platforms (e.g., NVIDIA Jetson series) to validate practical deployment feasibility in clinical settings.

## Conclusion

In this study, YOLO-LS, an enhanced framework derived from YOLO11n-seg, is presented for the robust recognition and segmentation of brain tumor MRI images. By synergistically integrating a ShuffleNet V1 lightweight backbone, the DySample dynamic upsampling mechanism, and the C3k2-PoolingFormer module, the proposed architecture effectively addresses the challenges of computational redundancy, feature loss, and boundary ambiguity. Empirical evaluations on the Figshare and Kaggle datasets demonstrate that YOLO-LS achieves a bounding box mAP50 of 0.953 ± 0.011 and a Dice coefficient of 0.910 ± 0.010. Significantly, the model attained a 95% Hausdorff Distance (HD95) of 4.35 ± 0.34 mm, reflecting statistically significant improvements in boundary delineation accuracy over state-of-the-art models like Swin-UNet and VM-UNet (paired t-test, *p* < 0.05). These gains were accompanied by a reduction in computational cost to 8.1 GFLOPs. Consequently, YOLO-LS establishes a favorable balance between computational efficiency and high-precision segmentation, demonstrating substantial potential for clinical deployment, with potential for deployment in resource-constrained clinical environments pending hardware-specific validation. Future investigations will prioritize multimodal data fusion and federated learning to further augment clinical utility.

## Data Availability

The datasets analyzed during the current study are available in the following public repositories: The Figshare brain tumor dataset is available at https://www.kaggle.com/datasets/ashkhagan/figshare-brain-tumor-dataset (as referenced in citations 31 and 32). The Kaggle brain tumor MRI dataset is available at https://www.kaggle.com/datasets/masoudnickparvar/brain-tumor-mri-dataset (as referenced in citation 33). No new datasets were generated in this study.
